# Risk factors associated with positional plagiocephaly in healthy Iranian infants: a case-control study

**DOI:** 10.22037/ijcn.v16i1.28524

**Published:** 2022-03-14

**Authors:** Babak SOLANI, Motahare TALEBIAN ARDESTANI, Homa BOROUMAND, Vahidreza OSTADMOHAMMADI, Mohammad HALLAJNEJAD, Mansour KASHANI ZADE, Amirhossein LOGHMAN, Ahmad TALEBIAN ARDESTANI

**Affiliations:** 1 Infectious Diseases Research Center, Kashan University of Medical Sciences, Kashan, Iran; 2 Department of Pediatrics, Faculty of Medicine, Kashan University of Medical Sciences, Kashan, Iran; 3 Skull Base Research Center, Loghman Hakim Hospital, Shahid Beheshti University of Medical Sciences, Tehran, Iran; 4 Department of Pathology, Faculty of Medicine, Kashan University of Medical Sciences, Kashan, Iran

**Keywords:** Positional plagiocephaly, infants, risk factor

## Abstract

**Objectives:**

Deformation of the skull by external forces in the absence of synostosis has been defined as positional plagiocephaly (PP). The aim of this investigation was to determine the risk factors of PP in healthy Iranian infants.

**Materials & Methods:**

This case-control study was performed on 300 healthy Iranian infants aged 8-12 weeks who were referred to the pediatric neurology clinic at Shahid Beheshti Hospital of Kashan. Plagiocephaly evaluations were done using Argenta’s scale.

**Results:**

Based on multivariate logistic regression analysis, there was a significant association between PP and male gender (OR=2.26; P=0.002), head circumference (OR=1.22; P=0.006), multiple pregnancy (OR=2.55; P=0.03), abnormal presentation in uterine (OR=2.18; P=0.02), primiparity (OR=2.43; P=0.003), and supine sleep position (OR=2.97; P<0.001). However, type of delivery, firmness of headrest, oligohydramnios, and prolonged labor were not correlated with PP.

**Conclusions:**

The current investigation supports the idea that head circumference, male gender, primiparity, multiple pregnancy, supine sleep position, and abnormal presentation in the uterine are correlated with a greater incidence of PP. Further investigations should be undertaken to understand PP and its related risk factors fully.

## Introduction

Deformation of the skull by external forces in the absence of synostosis has been defined as positional plagiocephaly (PP) (1). This condition is of concern because the related changes and deformities, including abnormal head shape, frontal bossing, facial dissymmetry, ear misalignment, and asymmetrical orbits, can be permanent (1). After the “Back to Sleep” campaign, the incidence rate of sudden infant death syndrome decreased by approximately 50% from 1992 to 2001. However, there was a significant increase in PP, currently a common problem faced by physicians (2). It can be developed in the uterus, during birth, or after birth. The postnatal form of PP is one of the most frequent abnormal findings in otherwise healthy infants (3). Based on prior reports, some of the well-known risk factors for PP in a normal child include supine sleep position, lack of tummy time, bottle propping, and common use of car seats or swings seats (4-6). Although supine sleep is a major risk factor for plagiocephaly, not all supine sleepers develop PP (7). Furthermore, multiple factors, such as gestational age, intrauterine position, assisted delivery, oligohydramnios, birth order, presentation at birth, gender, ethnicity, infant neck disorders, developmental delay, and different infant care practices, may cause this problem (8).

Even if the prognosis of plagiocephaly is good, the deformities are persistent if not managed as soon as possible, with psychosocial complications (9, 10). Infants with plagiocephaly might be less active than healthy matched controls (2). Some researchers have also indicated a relationship between PP and developmental delay, most frequently in motor functions and language (10). Thus, developmental evaluation is suggested as a part of the treatment of infants with this problem (11). Moreover, if immediately treated, fewer infants will require helmet therapy or physical therapy, decreasing discomforts and losing money and time for the health system (12).

Although there is enough knowledge regarding the risk factors associated with PP in infants with developmental delay or other diseases, less is recognized about why PP occurs in healthy infants. In the current study, to better understand, we investigated some probable risk factors associated with PP in healthy Iranian infants.

## Materials & Methods

The present case-control study was designed to determine the risk factors of PP. Ethics approval was received from the Kashan University of Medical Sciences (KAUMS) Research Ethics Board (Kashan, Isfahan) on June 3, 2017. Healthy full-term infants (gestational age > 37 weeks) aged 8-12 weeks who were referred to the pediatric neurology clinic at Shahid Beheshti Hospital (Kashan, Iran) affiliated to KAUMS were included. Patients with neurological disorders, malformations, and craniosynostosis were excluded from this investigation. 173 infants with PP and 150 healthy controls during the study period were referred to the pediatric neurology clinic. Thirteen infants were excluded because of exclusion criteria (three patients with torticollis, six patients with congenital malformations, and four patients with neurologic problems), and ten patients were excluded because of missing information. After obtaining informed consent, we considered two methods to collect data: 1) Parents were requested to fill a questionnaire assessing risk factors of PP 2) Plagiocephaly evaluations using Argenta’s scale (13) were done by the main author (AT). We categorized risk factors in three groups as follows: 1) Pregnancy-related Factors: parity, multiple pregnancy, prolonged labor, type of delivery, presentation in uterine, and oligohydramnios; 2) Infant factors: gender, head circumference at birth; and 3) Postnatal factors: supine sleep position and firmness of headrest.

 **Statistical analysis**

Data were analyzed by the univariate logistic regression method for each risk factor. Odds ratios, P-values, and 95% CIs were calculated for every risk factor. At last, risk factors with a P-value <0.25 in the univariate regression method were analyzed as independent factors in the multivariate logistic regression method. A P-value < 0.05 was considered significant. All analyses were performed using SPSS software version 16 (Chicago, Illinois, USA).

## Results

Based on univariate logistic regression analysis, there was a significant association between PP and male gender (OR=2.42; P<0.001), head circumference (OR=1.26; P<0.001), multiple pregnancy (OR=3.21; P=0.003), abnormal presentation in uterine (OR=2.66; P=0.001), primiparity (OR=2.35; P=0.001), and supine sleep position (OR=3.07; P<0.001). However, type of delivery, firmness of headrest, oligohydramnios, and prolonged labor were not correlated with PP. 

We used multivariate logistic regression to analyze factors with P<0.25 in the univariate logistic regression method, including male gender, head circumference, multiple pregnancy, abnormal presentation in uterine, supine sleep position, and primiparity. Patients with PP were 2.26 times more likely to be male (P=0.002) or 1.22 times to have larger head circumference (P=0.006). In assessing risk factors related to pregnancy, cases had a 2.55 times greater chance to have a multiple pregnancy (P=0.03) and showed 2.18 greater odds of abnormal presentation than the control group (P=0.02). PP was not associated with a higher risk of prolonged labor. Among risk factors related to postnatal caring, patients and healthy individuals showed significant differences in sleep position with a higher risk of PP in the supine position (OR=2.97; P<0.001). Table 1 shows the results of univariate and multivariate logistic regression analyses.

## Discussion

Our finding indicated six factors associated with PP. These factors were male gender, head circumference, multiple pregnancy, abnormal presentation in the uterine, primiparity, and supine sleep position. Oligohydramnios, prolonged labor, firmness of headrest, and delivery type showed significant association with PP.

***1. Factors related to pregnancy***

The occurrence of plagiocephaly in multiple pregnancy may be due to an increase in uterine size (14, 15). Another explanation is that multiple pregnancy can lead to greater uterine tension by increasing abnormal presentations risks, such as breech or transverse presentation (16). As we know, multiple pregnancy is also a risk factor for preterm labor, which is a potential risk factor for PP according to Littlefield et al. (16), Kane et al.(17), and Gardner et al.(18). Also, premature newborns have less activity and delayed motor function than term neonates, which can induce PP (19, 20).

This study confirms that primiparity is associated with a greater risk of PP. In accordance with our results, previous studies have demonstrated a significant association between PP and primiparity (4, 19, 21). A possible explanation for these results is that the pelvic muscles in primiparous mothers have higher strength than multiparous ones, which limits fetus movements in the uterine (16, 22-24). We considered cephalic presentation a normal presentation, and other presentations, such as transverse and breech, were considered abnormal presentations. There are several possible explanations for this association. This association may be related to a higher risk of breech presentation in multiple pregnancies, which is a known risk factor for PP (19). Also, some researchers believe that uterine presentation is associated with postnatal head position preferences during sleep (25, 26). According to our findings, no significant correlation was found between oligohydramnios and PP. This supports evidence from a study conducted by Oh et al. (27), but our findings are in contrast with those of McKinney et al.(28). Finally, delivery type and prolonged labor were not associated with PP in our study.

***2. Infant factors***

Our findings indicated that the male gender was remarkably associated with PP, which is in agreement with prior studies. Male gender is a well-known risk factor for developing PP (4, 17, 19, 21, 27, 29). This association may be related to greater head circumferences and less flexibility in male newborns (17, 30-32). Greater head in male fetuses is more vulnerable to deformation during labor (33, 34). Another explanation for this is that the male head grows faster after birth, resulting in a higher PP incidence in male neonates (29, 35). The current study found that higher head circumference was related to a greater chance of developing PP. Although our results differ from those of Glasgow et al. (36), they are consistent with those obtained by Seoane et al. (37).

***3. Postnatal factors***

We found a significant association between PP and supine sleep position. These results are in line with those of previous studies indicating the supine sleep position as a significant risk factor for developing PP (4, 21, 29, 36, 38, 39). One explanation for these results is that the infant’s head receives more pressure on its posterior surface in the supine position (40). On the other hand, when an infant sleeps in a prone position, forces spread over face structures and result in lower pressure (41). Moreover, it is reported that infants who sleep in a prone position are more active, which results in earlier development of motor functions (40, 42). Sudden infant death syndrome (SIDS) is a remarkable cause of unexpected death among infants. However, the notable point is that the prone sleep position is a well-known risk factor for developing SIDS; thus, physicians suggest sleeping in a supine position to prevent SIDS, which can lead to PP (43). Therefore, several recommendations are given by pediatricians to prevent both PP and SIDS, such as changing the head position regularly when putting the infant down to sleep, increasing tummy time, and decreasing the time sating in car seats, cots, and bouncers (21, 38, 44, 45). In this investigation, we found no significant relationship between PP and firmness of headrest, which is in accordance with the findings of Hutchison et al.(38).

**Table 1:** Risk factors of positional plagiocephaly

Risk factors	Univariate OR (95% CI)	P-value	Multivariate OR^*^ (95% CI)	P-value
**Delivery** **type (cesarean)**	1.22 (0.74-2.04)	0.43	**-**	
**Male gender**	2.42 (1.50-3.91)	<0.001	2.26 (1.33-3.83)	0.002
**Head circumference**	1.26 (1.11-1.43)	**<0.001**	1.22 (1.06-1.40)	**0.006**
**Firmness of headrest**	1.31 (0.72-2.37)	0.36	**-**	
**Multiple pregnancy **	3.21 (1.50-6.88)	0.003	2.55 (1.12-5.81)	0.03
**Abnormal presentation in uterine**	2.66 (1.50-4.69)	0.001	2.18 (1.16-4.13)	0.02
**Prolonged labor**	1.09 (0.48-2.48)	0.84	**-**	
**Supine sleep** **position**	3.07 (1.90-4.94)	<0.001	**2.97 (1.77-5.01)**	**<0.001**
**Primiparity**	2.35 (1.39-3.97)	0.001	2.43 (1.36-4.35)	0.003
**Oligohydramnios**	1.26 (0.33-4.78)	0.74	-	

* Adjusted for confounding variables.

## Limitations

One of the limitations of this study was the low number of participants. Another limitation was that we did not evaluate other risk factors, such as birth weight, mother’s education, parents' occupation, etc. This issue should be considered in the interpretation of our findings.

## Conclusions

The current investigation supports the idea that head circumference, male gender, primiparity, multiple pregnancy, supine sleep position, and abnormal presentation in the uterine are correlated with a greater incidence of PP. These findings extend our knowledge of PP and its related risk factors, which is crucial to identifying infants with a greater risk of PP. Further investigations should be undertaken to understand PP and its related risk factors fully.

## Author’s contribution

BS, MF, MT-A, MK-Z, AL, and AT-A contributed to the conception, design, data collection, and drafting of the manuscript. VO and HB contributed to statistical analysis, drafting, and revision of the manuscript. AT-A supervised the study. All authors approved the paper for submission.

## Conflicts of interest

None. 
